# 537. Implementation of a Workflow for COVID-19 Monoclonal Antibody Infusions at a Veterans Affairs Medical Center

**DOI:** 10.1093/ofid/ofab466.736

**Published:** 2021-12-04

**Authors:** Pratish C Patel, Ayne Adenew, Angela McKnight, Kevin Jeng, Angelike P Liappis

**Affiliations:** 1 Washington DC VA Medical Center, Potomac, Maryland; 2 Washington DC Veterans Affairs Medical Center, Washington, DC

## Abstract

**Background:**

In the setting of the global pandemic due to COVID-19, high-risk patients with mild to moderate disease were identified as a group who would benefit from COVID-19 monoclonal antibody (mAB) treatment to mitigate progression to severe disease or hospitalization. The U.S. Food and Drug Administration (FDA), under Emergency Use Authorizations (EUA) approved multiple COVID-19 mAB therapies with specific criteria for eligibility of candidates, documentation of discussion with patients, and reporting of all errors and serious adverse events.

**Methods:**

A cross discipline working group implemented a mAB clinic at complexity level 1a VA Medical Center in metropolitan Washington, D.C. through collaboration of personnel committed to patient care. The team successfully persuaded hospital leadership to provide space and leveraged technologies for rapid communication and dissemination of education. A stewardship driven medical center wide surveillance system rapidly identified outpatients for screening; primary care and ED providers were engaged through various electronic methods of education, including email, web-based team communication, intranet webpages and other electronic modalities. Within the EMR, an order panel was implemented to assure that the key requirements of the EUA were met and the provider was guided to the appropriate mAB, nursing, and PRN rescue medication orders.

**Results:**

Of over 17,000 COVID-PCR tests were performed at our medical center, 198 outpatients were screened and 16 received COVID-19 mAB infusions between January 2, 2021 to May 31, 2021. One patient experienced a reaction requiring the infusion to be stopped and supportive medications to be administered; there were no long-term sequalae reported as a result of this event.

**Conclusion:**

A multidisciplinary collaboration is well suited to implement innovative processes and policies for novel therapies in the middle of a pandemic. An agile workflow, regular communications between members of the workgroup, and commitment of institutional leadership helped facilitate the changes necessary to provide our patients the opportunity to receive potentially life-saving therapies.

**Disclosures:**

**All Authors**: No reported disclosures

